# Mechanotransduction Activates Microglia and Impairs Phagocytosis in Stiff Amyloid‐β Plaques

**DOI:** 10.1002/advs.202503389

**Published:** 2025-05-23

**Authors:** Yulin Liu, Junjie Zhang, Yuxiang Zhao, Feixiang Fang, Siyu Zhang, Qiqi An, Jian Zhuang, Feng Xu, Fei Li

**Affiliations:** ^1^ The Key Laboratory of Biomedical Information Engineering of Ministry of Education School of Life Science and Technology Xi'an Jiaotong University Xi'an 710049 P. R. China; ^2^ Bioinspired Engineering and Biomechanics Center (BEBC) Xi'an Jiaotong University Xi'an 710049 P. R. China; ^3^ Key Laboratory of Education Ministry for Modern Design Rotor‐Bearing System School of Mechanical Engineering Xi'an Jiaotong University Xi'an 710049 P.R. China

**Keywords:** Alzheimer's disease (AD), amyloid‐β (Aβ) plaques‐associated microenvironment, microglia, scanning electrochemical microscopy (SECM), scanning ion conductance microscopy (SICM)

## Abstract

In Alzheimer's disease (AD), microglia are activated by mechanical and biochemical cues in the amyloid‐β (Aβ) plaque‐associated microenvironment, causing neuroinflammation. While the impact of Aβ stiffness on microglial activation and the dynamic interplay between inflammation and phagocytosis remain unclear. Here, an in vitro Aβ plaque‐associated microglia microenvironment model is built and investigated how the stiffness of Aβ plaques triggers microglial activation via the PIEZO1 mechanotransduction pathway. Scanning electrochemical microscopy and scanning ion conductance microscopy are employed to in situ monitor reactive oxygen species release, membrane permeability, and phagocytic activity of microglia. It is found that Aβ stiffness drives early microglial activation, forming an oxidative‐stressed microenvironment that impairs the membrane integrity of microglia. And the antioxidant‐resveratrol effectively improves the phagocytosis dysfunction of the impaired microglia. This work reveals the complex interplay among mechanical cues, neuroinflammation, and phagocytic dysfunction in microglia and suggests potential therapeutic strategies targeting microglial dysfunction in AD.

## Graphical abstract

The study constructs an in vitro Aβ plaque‐associated microglia model and uses SECM and SICM to in situ monitor changes in ROS levels, membrane permeability, and phagocytosis of microglia during Aβ clearance. It is found out that Aβ stiffness triggers microglial activation via the PIEZO1 mechanotransduction pathway, causing neuroinflammation and phagocytic dysfunction.

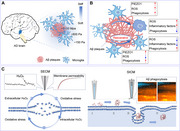

## 1 Introduction

Alzheimer's disease (AD) is a destructive neurodegenerative disorder and the foremost cause of dementia globally, affecting millions and imposing substantial social and economic burdens on healthcare systems.^[^ ^1^ ^]^ A pathological feature of AD is the accumulation of amyloid‐β (Aβ) peptides, which aggregate to form insoluble and stiff fibrillary plaques in the brain's extracellular matrix.^[^ ^2^, ^3^ ^]^ These Aβ plaques disrupt neuronal function and initiate a cascade of neuroinflammatory responses, ultimately leading to synaptic dysfunction and neuronal loss.^[^ ^4^ ^]^

Microglia, the macrophage‐like immune cells of the central nervous system, play a crucial role in AD by mediating both neuroprotective and neurotoxic effects.^[^ ^5^ ^]^ They are essential for maintaining neural homeostasis through the phagocytosis of cellular debris and the modulation of inflammatory responses.^[^ ^6^ ^]^ Upon encountering Aβ plaques, microglia become activated and migrate toward these deposits.^[^ ^7^ ^]^ While this activation is initially protective, chronic activation leads to the overproduction of pro‐inflammatory factors and reactive oxygen species (ROS), exacerbating neuronal damage.^[^ ^8^, ^9^ ^]^ Notably, the mechanical stiffness of Aβ plaques, exceeding 1 MPa, has been identified as a critical factor in triggering microglial activation via mechanotransduction pathways.^[^ ^9^ ^]^ Despite extensive research on the biochemical interactions between microglia and Aβ, the mechanistic understanding of how the mechanical properties of Aβ plaques influence microglial activation and neuroinflammation remains incomplete.

Emerging evidence underscores the significance of mechanosensitive ion channels, such as PIEZO1, in mediating cellular responses to mechanical stimuli.^[^ ^10^, ^11^ ^]^ Activation of PIEZO1 facilitates calcium (Ca^2+^) influx, initiating downstream signaling cascades that modulate inflammatory responses.^[^ ^12^ ^]^ However, the specific role of PIEZO1 in microglial activation induced by the mechanical stiffness of Aβ plaques has not been fully elucidated. Furthermore, enhancing microglia‐mediated Aβ clearance is a promising therapeutic strategy for AD.^[^ ^13^ ^]^ Yet, the interplay among mechanical cues, oxidative stress, and phagocytic dysfunction in microglia during the Aβ clearance process remains poorly understood. Addressing these gaps is crucial for identifying novel therapeutic targets and refining strategies to mitigate AD progression.

The early activation of Aβ plaque‐associated microglia can influence the functions of surrounding non‐activated microglia by altering the local neuroinflammatory microenvironment,^[^ ^14^ ^]^ there is still an unmet need for in situ monitoring of these rapid changes and their impact on microglial dysfunction near Aβ plaques. Scanning electrochemical microscopy (SECM) and scanning ion conductance microscopy (SICM) are two kinds of electrochemical principle‐based advanced scanning probe microscopy techniques that utilize micro/nanoelectrodes or nanopipettes to record faradaic or ionic currents of species in solution.^[^ ^15^, ^16^ ^]^ These two techniques offer noninvasive measurements with high temporal and spatial resolutions, making them ideal for dynamically tracking various cellular processes and species at the single‐cell level,^[^ ^17^, ^18^ ^]^ and have been successfully applied to study neuronal functions, including exocytosis,^[^ ^19^ ^]^ cellular membrane integrity,^[^ ^20^ ^]^ oxidative stress state,^[^ ^21^ ^]^ ion channel^[^ ^22^ ^]^ and cellular membrane structure.^[^ ^23^ ^]^ The integration of SECM and SICM thus provides a robust approach for monitoring the dynamic changes in microglial dysfunction, neuroinflammation, and phagocytic activity during the Aβ clearance process.

**Scheme 1:**
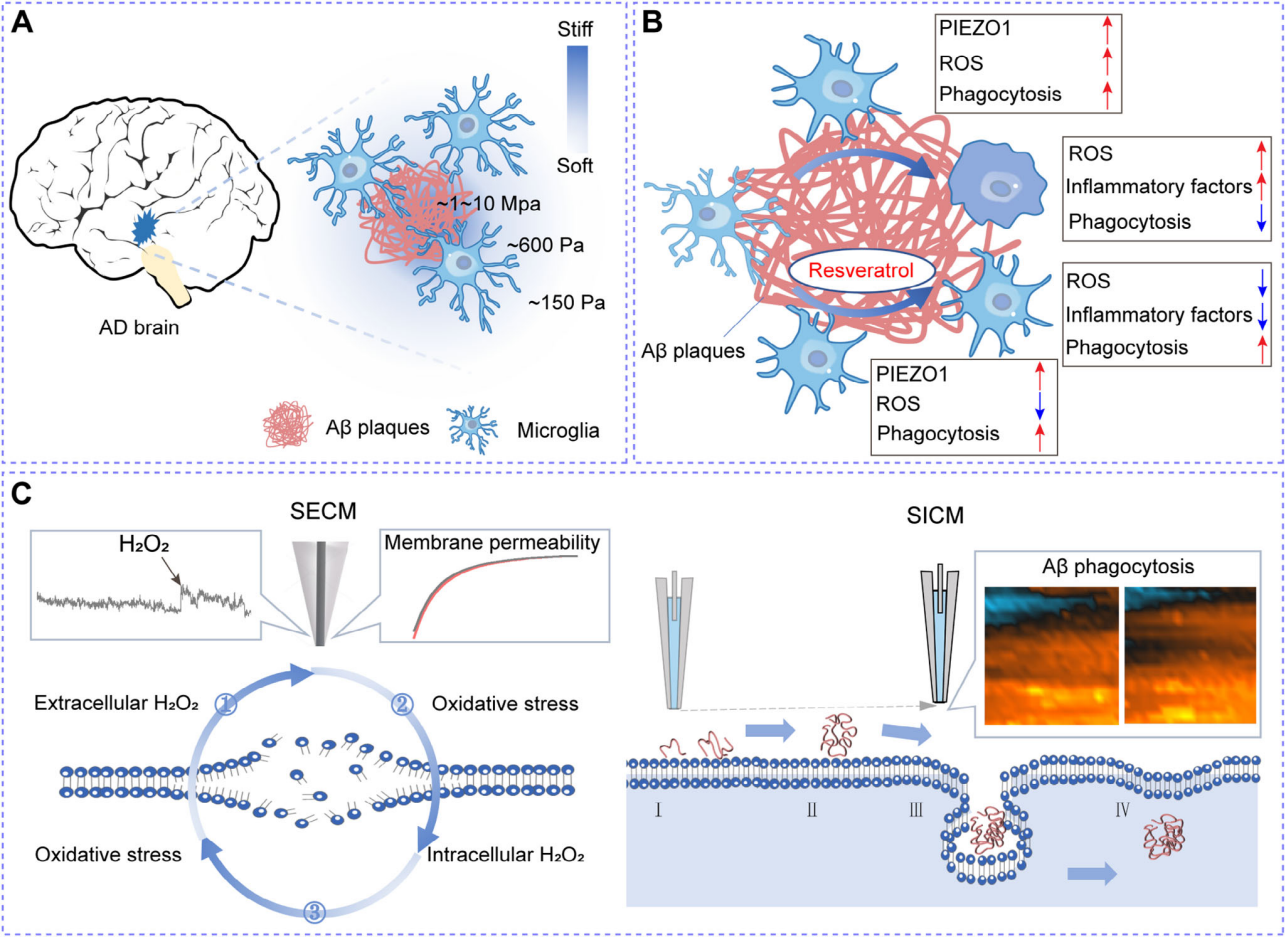
Schematic diagram of the investigation of microglial activation in the Aβ clearance process. A) Illustration of microglia in response to Aβ plaques in the brain; B) Schematic diagram of the activation process of Aβ plaque‐associated microglia and functional change of microglia during the Aβ clearance process; C) In situ monitoring of changes in H_2_O_2_ levels, membrane permeability, and Aβ phagocytosis of microglia in the Aβ clearance process using SECM and SICM.

In this study, we propose that the stiffness of Aβ plaques activates microglia via the PIEZO1 mechanosensing pathway, leading to early activation and neuroinflammation, which in turn impair microglial phagocytic function. To test this hypothesis, we first developed an in vitro Aβ plaque‐associated microenvironment by culturing BV2 cells on polyacrylamide (PA) gels deposited with Aβ plaques that mimic the mechanical stiffness of AD brain tissue. Then we used SECM and fluorescence microscopy to monitor the oxidative stress and cellular responses. We find that the mechanical stiffness of Aβ plaques activates microglia via the PIEZO1 pathway, leading to early extracellular hydrogen peroxide (H_2_O_2_) production before intracellular ROS accumulation and pro‐inflammatory factor release (**Scheme** **1**). Furthermore, we demonstrate that resveratrol, a potent antioxidant, mitigates oxidative stress, reduces neuroinflammation, and preserves microglial phagocytic activity by maintaining the expression of Triggering Receptor Expressed on Myeloid cells 2 (Trem2), a key modulator of phagocytosis. Additionally, SICM provides real‐time observations of microglial interactions with Aβ oligomers on the cell membrane, offering novel insights into the mechanisms of Aβ clearance. Collectively, our study elucidates a mechanotransduction‐mediated pathway of microglial activation induced by Aβ plaque stiffness and highlights the therapeutic potential of antioxidants like resveratrol in modulating microglial function. These findings contribute to our better understanding of AD pathogenesis and suggest new strategies for developing treatments targeting microglial dysfunction.

## 2 Results

### 2.1 An In Vitro Aβ Plaque‐Associated Microenvironment Induces Microglial Extracellular H_2_O_2_ Production Preceding Intracellular ROS Accumulation and Pro‐Inflammatory Factor Generation

**Figure 1:**
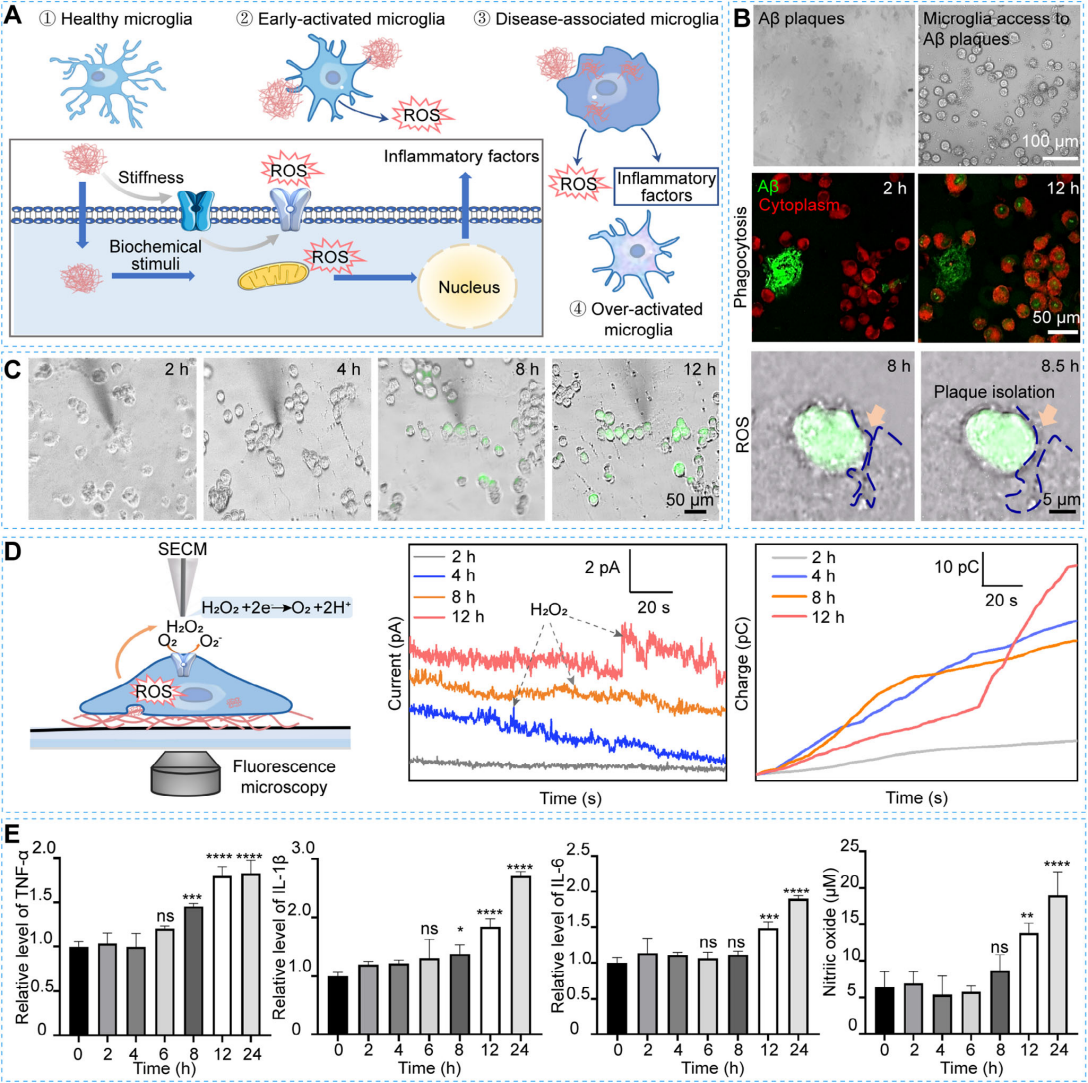
Aβ plaques‐associated microenvironment model of microglia and characterization results of microglial activation on 575.7 Pa PA gel during the Aβ clearance process. A) Schematic diagrams of microglial activation during Aβ clearance process; B) Construction of an in vitro Aβ plaque‐associated microenvironment induces microglial activation; C) Fluorescence images of intracellular ROS levels in BV2 cells on 575.7 Pa PA gel during Aβ clearance process; D) In situ monitoring of extracellular H_2_O_2_ level changes of a microglia using SECM, and the obtained current–time curves and charges of extracellular H_2_O_2_ of BV2 cells on 575.7 Pa PA gel after incubation of Aβ plaques for 2, 4, 8, and 12 h, respectively; E) Characterization results of relative TNF‐α, IL‐6, IL‐1β expressions and concentrations of NO of BV2 cells after incubation with Aβ plaques for 0, 2, 4, 6, 8, 12, and 24 h, respectively (*n* = 3). Statistical significance: *ns*, no significant difference, *p < 0.05, **p < 0.001, ***p < 0.0005, and ****p < 0.0001 (one‐way ANOVA).

To mimic the stiffness of brain tissue in the vicinity of Aβ plaques (≈600 Pa),^[^ ^9^ ^]^ we prepared the PA gels with the stiffness of 575.7 ± 82.7 Pa (Figure S1, Supporting Information). To simulate the Aβ plaque‐associated microenvironment and the process of Aβ clearance by microglia at the early, middle, and late stages of Aβ pathology, we incubated Aβ plaques onto the PA gels and seeded BV2 cells on the PA gels for incubation periods of 2 to 12 h (**Figure** **1**A). We used laser confocal microscopy to assess microglial activation during Aβ clearance (Figure 1B), demonstrating that Aβ plaques are stably deposited on the PA gels and microglia cluster around these plaques to eliminate Aβ. Additionally, the BV2 cells exhibit phagocytic activity by internalizing and clearing Aβ, alongside an oxidative stress state characterized by continuous ROS production and plaque isolation during the Aβ clearance process (Figure 1B). These observations align with previous reports,^[^ ^7^, ^24^ ^]^ and indicate that microglia convert into an activated state during Aβ clearance, leading to alterations in oxidative stress and neuroinflammatory states.^[^ ^25^ ^]^

ROS, the pro‐inflammatory signaling molecules, play a crucial role in immune responses under oxidative stress and neuroinflammation.^[^ ^21^ ^]^ Extracellular ROS, in particular, acts as an upstream signal for intracellular inflammatory cascades, significantly contributing to microglial activation throughout AD progression.^[^ ^24^ ^]^ To characterize the oxidative stress and neuroinflammatory state of microglia during Aβ clearance, we used SECM combined with fluorescence microscopy to in situ monitor extracellular H_2_O_2_ levels and intracellular ROS levels in BV2 cells cultured on 575.7 Pa PA gels after incubation with Aβ plaques for 2, 4, 8, and 12 h (Figure 1C,D). After 4 h of incubation with Aβ plaques, fluorescence imaging shows no significant intracellular ROS signal in BV2 cells (Figure 1C). However, the SECM measurements reveal dramatically enhanced extracellular H_2_O_2_ oxidation currents with periodic fluctuations (Figure 1D; Figure S2, Supporting Information). These results indicate that extracellular H_2_O_2_ production precedes intracellular ROS accumulation, suggesting a prioritized extracellular response in microglial activation.

We further monitored changes in both intracellular ROS and extracellular H_2_O_2_ levels in BV2 cells after 8 and 12 h of incubation with Aβ plaques. We observe that fluorescence signals for intracellular ROS in BV2 cells increase significantly with prolonged incubation time (Figure 1C). The H_2_O_2_ oxidation currents after 12 h are significantly higher than those after 4 and 8 h, while no significant difference is observed between the 4 and 8 h (Figure 1D). These results indicate that intracellular ROS levels in BV2 cells increase markedly during the 4–8 h period of Aβ phagocytosis, whereas extracellular ROS levels remain relatively show no obvious change, possibly due to that the extracellular ROS is scavenged by surrounding microglia.^[^ ^24^ ^]^ After 12 h of incubation, both intracellular and extracellular ROS levels in BV2 cells increase, likely due to the disruption of redox balance caused by sustained Aβ phagocytosis. This imbalance contributes to the formation of extracellular oxidative stress, disturbing nervous system homeostasis.^[^ ^25^ ^]^

To further quantify the changes in the extracellular H_2_O_2_ levels during the Aβ clearance process, we derived the charges of the extracellular H_2_O_2_ of BV2 cells by time‐integrating the H_2_O_2_ oxidation current signals after incubation with Aβ plaques for 2, 4, 8, and 12 h. The charges are 7.4 ± 2.4, 39.1 ± 6.9, 40.4 ± 5.8, and 57.5 ± 5.6 pC, respectively (Figure 1D). These results demonstrate that extracellular H_2_O_2_ levels increase with prolonged incubation time, indicating a shift from sustained to excessive ROS levels, which can trigger a pro‐inflammatory response during Aβ clearance.^[^ ^24^ ^]^

Next, we assessed pro‐inflammatory factor production in BV2 cells during Aβ clearance by measuring tumor necrosis factor‐alpha (TNF‐α), interleukin‐1 beta (IL‐1β), interleukin‐6 (IL‐6), and nitric oxide (NO) levels. We observe no significant changes in TNF‐α, IL‐1β, IL‐6, and NO concentrations after 4 h of incubation (Figure 1E), indicating that productions of these pro‐inflammatory factors occur after extracellular H_2_O_2_ production. After 8 h of incubation, levels of these pro‐inflammatory factors begin to increase gradually. Following 12 h of incubation, we observe significant increases in the relative levels of TNF‐α, IL‐1β, IL‐6, and NO concentrations in BV2 cells by 1.8 ± 0.10, 1.84 ± 0.14, 1.48 ± 0.09, and 7.45 ± 1.31 µm, respectively (Figure 1E). These findings demonstrate that with Aβ plaques, both pro‐inflammatory factors and ROS levels in BV2 cells significantly upregulate with prolonged incubation time, with extracellular H_2_O_2_ production preceding the increase of other pro‐inflammatory factors.

Collectively, our findings demonstrate that extracellular H_2_O_2_ production by microglia precedes intracellular ROS accumulation and pro‐inflammatory factor production during the early stages of Aβ clearance. This early extracellular H_2_O_2_ production characterizes early microglial activation, serving as a trigger between neuroinflammation and early Aβ pathology.^[^ ^26^ ^]^

### 2.2 Stiffness of Aβ Plaques Triggers Microglial Activation via PIEZO1 Mechanosensing Pathway, Leading to Neuroinflammation

**Figure 2:**
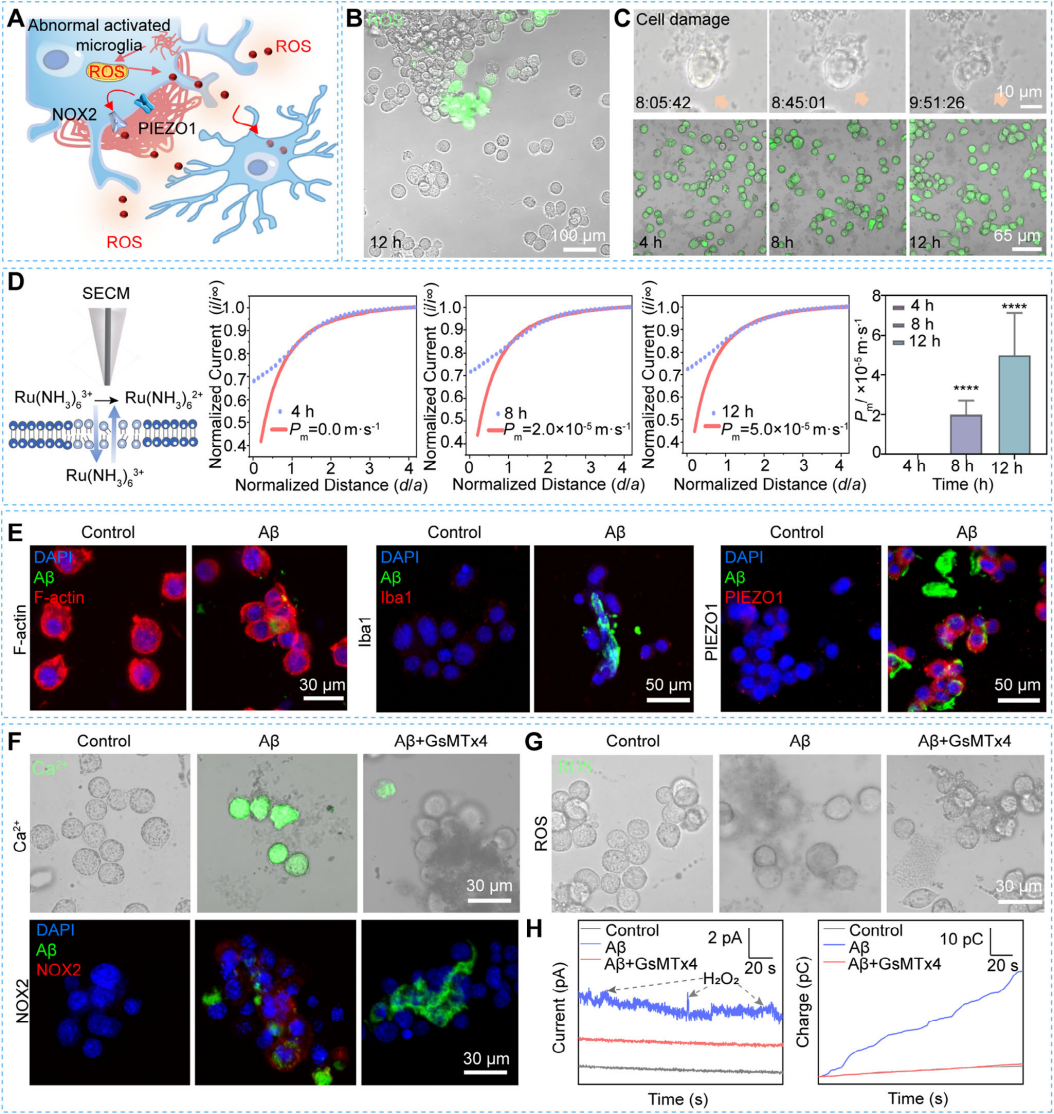
Characterization results of intracellular ROS levels, membrane permeability, and PIEZO1‐related mechanotransduction pathway of BV2 cells on 575.7 Pa PA gels during the Aβ clearance process. A) Schematic diagram of a local inflammation microenvironment created by Aβ plaque‐induced microglial activation; B) Fluorescence images of intracellular ROS levels in BV2 cells after incubation of Aβ plaques for 12 h; C) Optical microscope images of BV2 cell after incubation of Aβ plaques for 8 h, and fluorescence images of living (green) and dead (red) BV2 cells after incubation of Aβ plaques for 4, 8, and 12 h, respectively; D) Schematic diagram of monitoring of membrane permeability of BV2 cells using SECM, the recorded approach curves of BV2 cells on the PA gels after incubation of Aβ plaques for 4, 8, and 12 h, respectively, and the statistical results of average *P*_m_ values of BV2 cells for different times (*n* = 9); E) Fluorescence images of F‐actin, Iba1, PIEZO1 in BV2 cells on the PA gels at 24 h and after incubation of Aβ plaques for 4 h; Fluorescence images of F) Ca^2+^ label, NOX2 and G) intracellular ROS levels in BV2 cells on the PA gels after incubation of Aβ plaques for 4 h without or with GsMTx4 (2.5 µm) treatment; H) Current–time curves and charges of extracellular H_2_O_2_ of BV2 cells on the PA gels after incubation of Aβ plaques for 4 h without or with GsMTx4 (2.5 µm) treatment, recorded by SECM. Statistical significance: ****p < 0.0001 (one‐way ANOVA).

The Aβ plaque‐induced microglial activation could create a localized inflammatory microenvironment (**Figure** **2**A).^[^ ^27^ ^]^ To investigate how Aβ plaque stiffness influences microglial activation and neuroinflammatory responses, we assessed the oxidative stress state and viability of BV2 cells located near and distant from Aβ plaques after a 12 h incubation period. We observe that the intracellular ROS levels are significantly elevated in BV2 cells surrounding the plaques compared to those farther away, which do not exhibit noticeable ROS fluorescence signals (Figure 2B). These results indicate that sustained Aβ phagocytosis by microglia induces ROS‐mediated oxidative stress, creating a localized extracellular oxidative environment that may lead to neuroinflammation and neurotoxicity in microglia near Aβ plaques.

To evaluate microglial cell damage during the Aβ clearance process, we monitored membrane integrity using optical microscopy after 9 h of incubation with Aβ plaques. We observe signs of microglial neurotoxicity during the Aβ clearance process, but there are no significant differences in overall cell viability after 4, 8, and 12 h of incubation as reflected from live/dead staining (Figure 2C). These findings suggest that traditional cell viability assays may not sensitively detect neurotoxicity in the context of Aβ clearance, potentially due to rapid microglial proliferation and efficient removal of cell debris.^[^ ^28^ ^]^ Therefore, we focus on changes in cell membrane permeability as a more accurate indicator of oxidative stress‐induced damage.^[^ ^29^ ^]^

To monitor membrane permeability changes in situ during the Aβ clearance process, we employed scanning electrochemical microscopy (SECM) using hydrophilic [Ru(NH_3_)_6_]Cl_3_ as a redox mediator (Figure 2D). We quantified membrane permeability by determining the permeability coefficient (*P*_m_) through fitting normalized experimental approach curves to theoretical models (Figure S3, Table S1, Supporting Information). Our SECM measurements reveal that after 4 h of incubation with Aβ plaques, the normalized approach curve aligns with the theoretical curve for *P*_m_ =  0.0 m·s^−1^, indicating intact membrane integrity. In contrast, after 8 and 12 h of incubation, *P*_m_ values increase to 2 × 10^−5^ and 5 × 10^−5^ m·s^−1^, respectively (Figure 2D). The plot of average *P*_m_ values versus incubation time demonstrates that membrane permeability begins to rise at 8 h, likely due to internalized Aβ damaging mitochondria and lysosomes. This damage leads to ROS release and extracellular H_2_O_2_ accumulation, which synergistically impairs cell membrane integrity.^[^ ^29^ ^]^ These SECM results suggest that oxidative stress and neuroinflammation induced during Aβ phagocytosis compromise microglial membrane integrity over time.

Furthermore, we observe that BV2 cells cluster around Aβ plaques after 4 h of incubation and exhibit elongated and polarized morphologies (Figure 2E). This morphological change may result from microglial activation driven by the physical stiffness of Aβ plaques rather than activation through internalized Aβ, which is known to increase Iba1 expression (Figure 2E). Previous studies have reported that PIEZO1 is the most highly expressed mechanosensitive ion channel in microglia compared to other mechanosensory ion channels.^[^ ^9^ ^]^ Additionally, the mechanotransduction pathway is linked to matrix stiffness‐dependent microglial activation, with increased expression of PIEZO1.^[^ ^10^, ^30^ ^]^ To determine whether Aβ plaque stiffness mediates microglial morphological changes via the PIEZO1 pathway, we examined PIEZO1 protein levels in BV2 cells cultured on 575.7 Pa PA gels with and without Aβ plaques using immunofluorescence staining. We find no significant PIEZO1 expression in BV2 cells cultured without Aβ plaques for 24 h. However, after incubation with Aβ plaques for 4 h, the PIEZO1 expression levels in BV2 cells increase by 2.18‐fold compared to controls (Figure 2E). Moreover, from the time‐course results of PIEZO1 expressions in BV2 cells after incubation of Aβ plaques for 4, 8, and 12 h, respectively, we find that the PIEZO1 expressions in BV2 cells exposed to Aβ plaques are significantly upregulated at 4 h compared to the cells without Aβ plaques. No significant differences are detected at 8 and 12 h during the Aβ clearance process (Figure S4, Supporting Information). These results suggest that Aβ plaque stiffness predominantly induces PIEZO1 expression, and this effect is not influenced by the duration of Aβ incubation.

Next, to validate the involvement of PIEZO1 at the stiffness‐mediated early microglial activation process and given that Ca^2+^ influx is crucial for PIEZO1‐mediated responses to mechanical stimuli and can enhance extracellular ROS production,^[^ ^30^ ^]^ we measured the Ca^2+^ influx in BV2 cells after incubation of Aβ plaques for 4 h (Figure 2F). We observe the elevated Ca^2+^ influx in BV2 cells after incubation of Aβ plaques, which correlates positively with the increased PIEZO1 expression in response to Aβ plaques. After inhibiting PIEZO1 with GsMTx4 (a typical inhibitor of PIEZO1), the Ca^2+^ influx in BV2 cells significantly reduced compared to the cells without adding GsMTx4, indicating that the Ca^2+^ influx in BV2 cells is dependent on PIEZO1 mechanotransduction. We also measured the expressions of NADPH oxidase 2 (NOX2), the main protein responsible for extracellular ROS generation, after incubation with Aβ plaques for 4 h without and with adding GsMTx4 (Figure 2F). The NOX2 expression is upregulated by ≈1.54‐fold in BV2 cells after 4 h of incubation with Aβ plaques compared to controls. This upregulation promotes electron transport from intracellular to extracellular spaces, catalyzing the conversion of O_2_ to O_2_⁻ and extracellular H_2_O_2_,^[^ ^31^, ^32^, ^33^ ^]^ thereby favoring extracellular over intracellular ROS production. After inhibition of PIEZO1 with GsMTx4, the NOX2 expression in BV2 cells incubated with Aβ plaques significantly reduced compared to the cells without adding GsMTx4 (Figure 2F). We do not observe a significant difference in the intracellular ROS signal in BV2 cells compared to the cells without adding GsMTx4 (Figure 2G). Furthermore, we utilized SECM to measure extracellular H_2_O_2_ levels of BV2 cells after incubating with Aβ plaques for 4 h without and with adding GsMTx4. We observe that the extracellular ROS levels of GsMTx4‐treated BV2 cells are significantly lower than the untreated cells (Figure 2H). Through integrating current signals over time, we obtain the charges of extracellular ROS levels without and with adding GsMTx4 as 35.6 ± 5.6 and 6.5 ± 2.4 pC, respectively. These results confirm the role of PIEZO1 as a mechanotransduction pathway at the early activation process and oxidative stress of microglia triggered by the Aβ plaques stiffness.

These findings indicate that the stiffness of Aβ plaques enhances extracellular ROS levels via the PIEZO1 mechanosensing pathway in microglia. Collectively, our results demonstrate that microglia undergo PIEZO1‐dependent early activation in response to Aβ plaque stiffness, leading to increased extracellular ROS production before Aβ phagocytosis occurs. This persistent extracellular oxidative stress, combined with internalized Aβ synergistically impairs cell membrane over time, contributing to neuroinflammation and neurotoxicity during the Aβ clearance process.

### 2.3 Resveratrol Mitigates Oxidative Stress and Sustains Microglial Phagocytic Activity during Aβ Clearance

**Figure 3:**
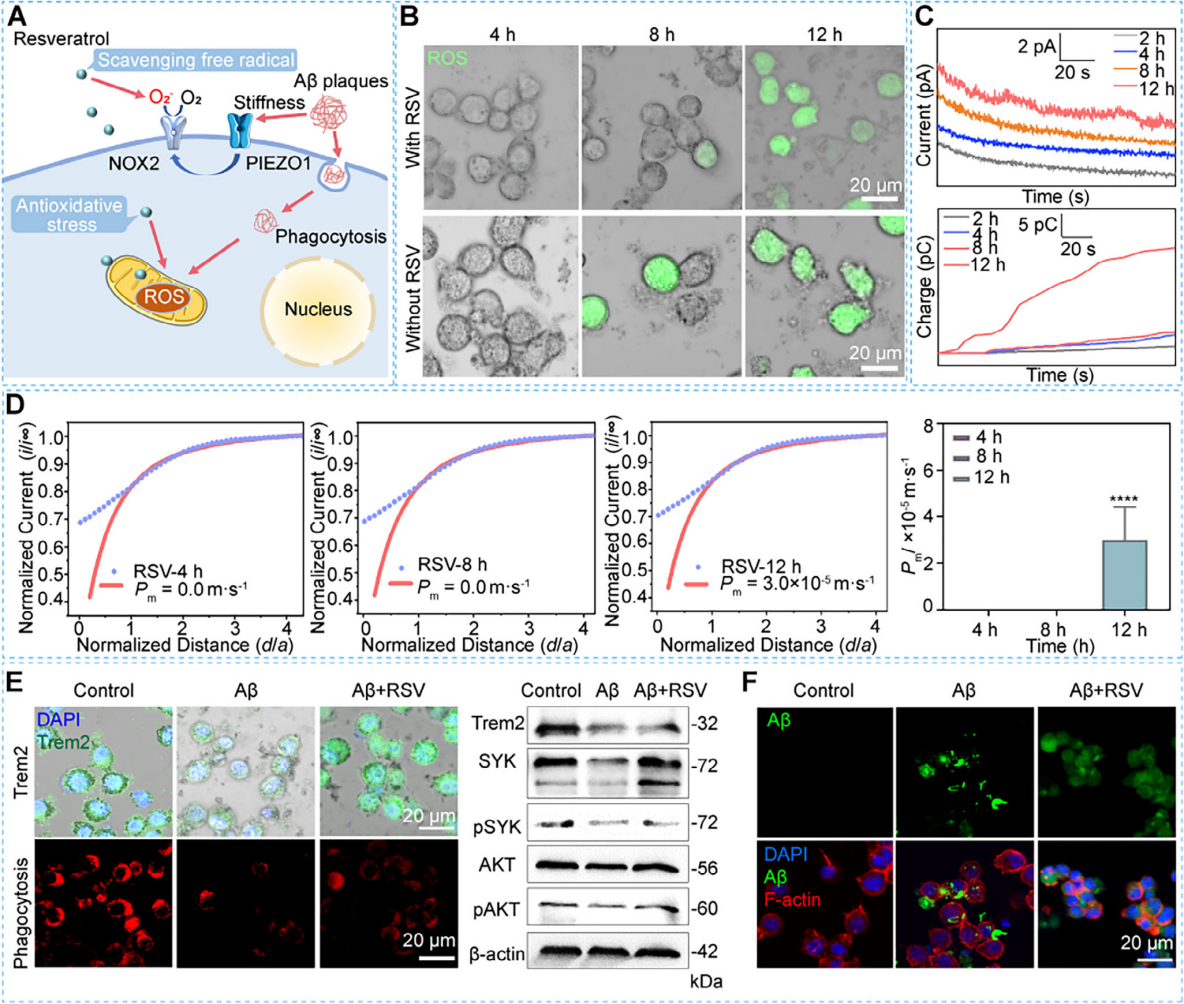
Characterization results of ROS levels, membrane permeability, and signaling pathway of Trem2 of BV2 cells on 575.7 Pa PA gels in the Aβ clearance process under RSV treatment. A) Schematic diagram of ameliorate oxidative stress and neuroinflammation in microglia by RSV; B) Fluorescence images of intracellular ROS levels in BV2 cells after incubation of Aβ plaques for 4, 8, and 12 h with and without RSV treatment, respectively; C) Current–time curves and charges of H_2_O_2_ oxidation of BV2 cells on the PA gels with Aβ plaques at 2, 4, 8, and 12 h under RSV treatment, respectively, recorded by SECM; D) SECM results of cell membrane permeability and statistical results of average *P*_m_ values of BV2 cells on the PA gels after incubation of Aβ plaques for 4, 8, and 12 h under RSV treatment, respectively, (*n* = 9); E) Relative Trem2, SYK, p‐SYK, AKT, p‐AKT expressions and phagocytic activity of BV2 cells on the PA gels with and without RSV treatment, respectively; F) Fluorescence images of intracellular FAM‐labeled Aβ in BV2 cells with and without RSV treatment. Statistical significance: ****p < 0.0001 (one‐way ANOVA).

Antioxidants are known to ameliorate oxidative stress and neuroinflammation in microglia, potentially delaying AD progression.^[^ ^13^, ^34^ ^]^ Resveratrol (RSV), a potent antioxidant with anti‐inflammatory properties, can penetrate blood–brain barrier and demonstrates neuroprotective efficacy along with mitochondrial repair function in inhibiting the development of neurological diseases (**Figure** **3**A).^[^ ^35^ ^]^ To determine whether RSV can alleviate oxidative stress and reduce the inflammatory burden on microglia during Aβ clearance, we treated BV2 cells with 10 µm RSV (Figure S5, Supporting Information) and measured both intracellular and extracellular ROS levels after incubating BV2 cells with Aβ plaques for 4, 8, and 12 h. We observe that at 4 h, there is no significant difference in intracellular ROS levels between RSV‐treated and untreated BV2 cells. However, at 8 and 12 h, intracellular ROS levels in RSV‐treated BV2 cells are significantly lower compared to those of untreated cells (Figure 3B). Furthermore, to investigate whether RSV has a mitochondrial repair function during the Aβ clearance process, we measured the ATP levels in BV2 cells incubated with Aβ plaques for 4, 8, and 12 h, respectively (Figure S6, Supporting Information). We observe that the ATP levels in BV2 cells incubated with Aβ plaques for 4 h is elevated, which can be owing to the increased metabolic demand from immune activation. While after incubation with Aβ plaques for 8 and 12 h, the ATP levels in BV2 cells are significantly lower than those for 4 h, which is likely due to mitochondrial dysfunction and increased oxidative stress as the phagocytic process progresses. In addition, after RSV treatment, the ATP levels in BV2 cells incubated with Aβ plaques for 4 and 8 h present no significant differences, while an increase for 12 h compared to those without RSV treatment. It indicates that RSV possesses mitochondrial protective properties and the ability to alleviate oxidative stress, contributing to sustaining metabolic demand on microglia during Aβ clearance. These results demonstrate that RSV can effectively reduce oxidative stress and exhibit mitochondrial repair function in microglia during the early and middle stages of Aβ clearance by scavenging ROS and sustaining ATP levels.

To assess whether RSV also decreases extracellular H_2_O_2_ production and prevents the formation of an oxidative extracellular microenvironment, we employed SECM to monitor extracellular H_2_O_2_ levels of BV2 cells in situ. After RSV treatment, we observe no significant H_2_O_2_ oxidation current signals outside BV2 cells at 2, 4, and 8 h of incubation with Aβ plaques. At 12 h, periodic fluctuations in H₂O₂ oxidation currents are present but are significantly lower than those without RSV treatment (Figure 3C). We quantified extracellular H_2_O_2_ levels by integrating the current signals over time, obtaining charges of 3.7 ± 2.1, 5.3 ± 2.2, 5.8 ± 1.1, and 21.1 ± 1.6 pC at 2, 4, 8, and 12 h, respectively (Figure 3C). These findings confirm that RSV treatment decreases extracellular H_2_O_2_ levels during the Aβ clearance process, aligning the trend of extracellular ROS levels with the reduced intracellular ROS observed earlier. Moreover, to investigate whether RSV affects PIEZO1 expression in microglia, we measured the PIEZO1 expression in BV2 cells after incubation with Aβ plaques for 4, 8, and 12 h, respectively, with RSV treatment (Figure S7A, Supporting Information). The obtained quantitative analysis results in Figure S7B (Supporting Information) show that the RSV treatment does not affect the PIEZO1 expression in BV2 cells during the Aβ clearance process, proving that RSV does not influence PIEZO1 expression of microglia in our case.

Next, we investigate whether RSV can mitigate neurotoxicity induced by persistent oxidative stress by measuring membrane permeability coefficients of BV2 cells after incubation with RSV for 4, 8, and 12 h using SECM. The *P*_m_ values are 0.0, 0.0, and 3 × 10^−5^ m·s^−1^ at 4, 8, and 12 h, respectively (Figure 3D). These results indicate that RSV prevents cell membrane destruction by scavenging ROS during the Aβ clearance process, thereby maintaining membrane integrity and reducing neurotoxicity.

Triggering receptor expressed on myeloid cells 2 (Trem2) plays a pivotal role in modulating microglial phagocytosis of Aβ, but its dysfunction is associated with disease progression and aging.^[^ ^36^ ^]^ To determine whether RSV influences Trem2 expression during the Aβ clearance process, we assessed Trem2 levels in BV2 cells after 12 h of incubation with Aβ plaques by immunofluorescence staining. We observe that Trem2 expression in BV2 cells decreases after 12 h of incubation with Aβ plaques compared to the cells without Aβ plaques (Figure 3E). Importantly, RSV treatment sustains Trem2 protein expression in BV2 cells exposed to Aβ plaques. To further investigate the underlying mechanism of RSV on Trem2, we performed western blot analysis to quantify Trem2 and its downstream signaling molecules (SYK, pSYK, AKT, and pAKT) in BV2 cells incubated with Aβ plaques for 12 h with and without RSV treatment. As shown in Figure 3E, Trem2 expression decreases in BV2 cells after 12 h of incubation with Aβ plaques compared to control cells without Aβ plaques. However, RSV treatment significantly increases Trem2 expression in BV2 cells exposed to Aβ plaques, which is consistent with the immunofluorescence data. Additionally, the western blot results of SYK, pSYK, AKT, and pAKT demonstrate that RSV upregulates the Trem2 downstream signaling pathway. These findings suggest that RSV activates the Trem2‐SYK‐PI3K/AKT signal transduction pathway, potentially improving microglial Aβ phagocytosis. Moreover, our results indicate that sustained Aβ phagocytosis leads to decreased Trem2 expression, potentially due to microglial aging, and that RSV can delay microglial aging to sustain Trem2 levels during Aβ clearance (Figure S8, Supporting Information).

Finally, to verify whether RSV enhances microglial phagocytic activity by sustaining Trem2 expression, we measured the phagocytic activity of microglia after 12 h of incubation with Aβ plaques, with or without RSV treatment, using phagocytic assay kits (Figure 3E). The RSV‐treated BV2 cells engulf more pHrodo particles, which fluoresce brightly in acidic phagosomal environments, than untreated cells (Figure 3E). Additionally, using FAM‐labeled Aβ plaques, we observe that the RSV‐treated BV2 cells are more likely to phagocytose aggregated FAM‐labeled Aβ plaques compared to those without RSV treatment (Figure 3F). These results demonstrate that RSV significantly upregulates microglial phagocytic activity during the Aβ clearance process.

Moreover, we investigated the effect of RSV treatment on pro‐inflammatory factor expression of BV2 cells after 12 h of incubation with Aβ plaques. We find that the expression levels of TNF‐α, IL‐6, IL‐1β, and NO concentrations of BV2 cells are lower in the RSV‐treated cells compared to those without RSV treatment (Figure S9, Supporting Information). These findings confirm that RSV exerts anti‐inflammatory effects on microglia during the Aβ clearance process, and sustains Trem2 expression to improve phagocytic activity by delaying microglial aging, and ultimately enhances the clearance of Aβ plaques compared to treatment without RSV.

Collectively, these findings demonstrate that RSV reduces both intracellular and extracellular oxidative stress, sustains Trem2 expression, and enhances phagocytic activity in microglia during Aβ clearance. By mitigating oxidative stress and maintaining microglial function, RSV effectively reduces the inflammatory burden on microglia, offering a promising therapeutic strategy for delaying AD progression.

### 2.4 Resveratrol Mitigates Oxidative Stress and Neuroinflammation, Thereby Enhancing Microglial Phagocytosis during the Late Stage of Aβ Clearance

**Figure 4:**
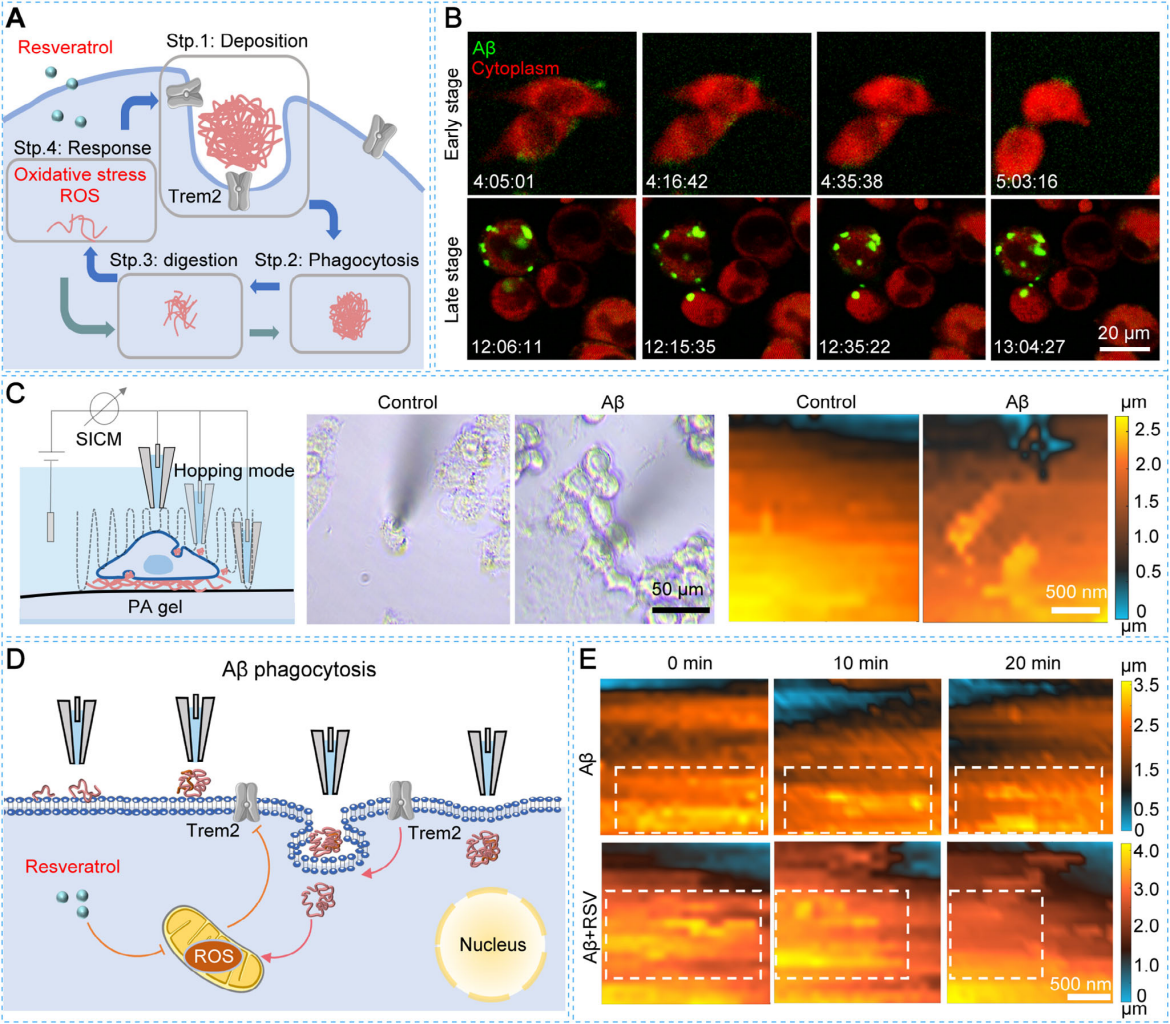
Monitoring of RSV‐enhanced microglial Aβ phagocytosis on a BV2 cell membrane on 575.7 Pa PA gels using fluorescence imaging and SICM. A) Schematic diagram of RSV enhances the change of microglia phagocytic activity at the Aβ clearance process; B) Fluorescence images of the phagocytic ability of BV2 cells at the early and late stages of Aβ clearance process; C) Schematic of SICM system for monitoring topography of microglial membrane surface with Aβ oligomers using a nanopipette as its probe and hopping mode, and the optical microscope images (left) and SICM images (right) of Aβ oligomers on a BV2 cell membrane without and with incubation of Aβ plaques for 12 h; D) Schematic illustration of SICM to in situ investigate microglial phagocytic process during Aβ clearance process; E) SICM time‐lapse images of change of microglial phagocytic activity at the late stage of Aβ clearance with and without RSV treatment.

At the late stage of AD, microglial neuroinflammation and neurotoxicity contribute to dysfunction in phagocytosis (**Figure** **4**A). This dysfunction is typically thought to impair the clearance efficiency of microglia for Aβ aggregates.^[^ ^37^ ^]^ However, the extent to which RSV enhances phagocytosis and influences the real‐time dynamics of Aβ oligomers on cell membrane surfaces during late‐stage phagocytic activity remains unclear. Therefore, monitoring changes in microglial phagocytic activity during this stage, alongside evaluating the potential antioxidant therapeutic effects of RSV, is essential for understanding the role of microglia in Aβ clearance and disease progression.^[^ ^38^ ^]^

To assess changes in microglial phagocytosis during the late stages of Aβ clearance, we employed fluorescence imaging within a 1 h timeframe to monitor Aβ clearance efficiency at both early and late stages. We observe that the BV2 cells cultured on 575.7 Pa PA gels after 4 h of incubation with Aβ plaques actively phagocytose FAM‐labeled Aβ and undergo morphological transformations (Figure 4B). In contrast, the BV2 cells after 12 h of incubation exhibit an over‐activated, rounded shape and display dynamic motion of Aβ oligomers on their membrane surfaces in real time, indicating decreased phagocytic activity at the late stage of Aβ clearance.^[^ ^14^ ^]^ However, detecting the dynamic changes of Aβ oligomers on living cell membranes in real‐time and with high resolution has consistently been an unmet requirement.

To in situ characterize the dynamic changes of Aβ oligomers on living cell membranes, we utilized SICM with a ≈30 nm‐in‐diameter nanopipette as its probe to characterize the cell surface changes during the microglial phagocytic process (Figure 4C). First, we used SICM to examine Aβ accumulation on the membranes of fixed BV2 cells after 12 h of incubation with and without Aβ plaques. We find that the surfaces of BV2 cells exhibit noticeable Aβ oligomers, whereas cells without Aβ plaques maintain smooth surfaces (Figure 4C). This indicates that Aβ plaques can be isolated and transferred to cell surfaces during the clearance process.

To monitor the dynamic phagocytic activity of living microglia at the late stage of Aβ clearance, we employed SICM to track real‐time changes in phagocytic behavior (Figure 4D). Aβ oligomers on the living BV2 cells surfaces appear more scattered and irregular compared to those on fixed cells. Additionally, the oligomers move continuously and dynamically on the cell membrane over a 20 min period (Figure 4E). This phenomenon may result from the greater membrane flexibility and dynamic properties of living microglia compared to fixed cells, as well as the ongoing process of Aβ accumulation on the microglial membrane.^[^ ^39^ ^]^

To further evaluate the efficacy of RSV treatment on microglial phagocytic ability in real time, we treated BV2 cells with RSV after 12 h of incubation with Aβ plaques and used SICM to assess Aβ uptake efficiency. The RSV‐treated BV2 cells exhibit more pronounced motion of Aβ oligomers on the cell membrane, smoother cell membranes, and a more migratory cell morphology, indicating faster dynamic processes of Aβ accumulation compared to the untreated cells (Figure 4E). Moreover, Aβ oligomers on the cell membrane are phagocytosed within 20 min in RSV‐treated BV2 cells, demonstrating that RSV enhances the phagocytic ability of microglia during the late stage of Aβ clearance.

These results indicate that microglia exhibit decreased phagocytic activity at the late stage of Aβ clearance. RSV treatment enhances the phagocytic ability of microglia during this stage, likely by mitigating oxidative stress and neuroinflammation. This suggests that RSV may be a promising therapeutic strategy to improve microglial function and potentially slow the progression of AD.

## 3 Discussion

### 3.1 Mechanical Stiffness of Aβ Plaques Triggers Early Microglial Activation via PIEZO1

**Figure 5:**
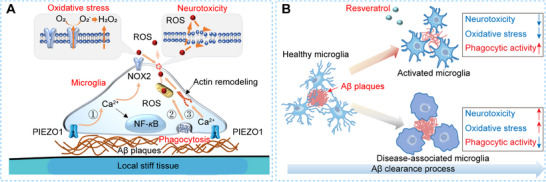
A) Scheme of mechanisms of stiffness of Aβ plaques and phagocytosis‐induced microglial activation accompanied by changes in oxidative stress, neurotoxicity during Aβ clearance process; B) RSV ameliorate the inflammatory burden of microglia in Aβ clearance process to alleviate neurotoxicity and improve the phagocytic activity.

In this study, we investigate how the mechanical stiffness of Aβ plaques influences microglial activation and the subsequent neuroinflammatory responses during Aβ clearance. We construct an in vitro mechanical microenvironment model that mimics the stiffness of brain tissue surrounding Aβ plaques, allowing us to observe the microglial Aβ clearance process under pathological conditions characteristic of AD. Using SECM combined with fluorescence labeling, we characterize changes in ROS levels and discover that extracellular ROS production by microglia is associated with their early activation. This activation is triggered by the stiffness of Aβ plaques, preceding the phagocytosis of Aβ by microglia. Our findings indicate that the mechanical stiffness of Aβ plaques upregulates the mechanosensitive ion channel PIEZO1, initiating a mechanotransduction pathway that leads to sustained ROS production and neuroinflammation (**Figure** **5**A). Consequently, the stiffness‐induced oxidative stress fosters an early activation state in microglia within the Aβ plaque‐associated microenvironment.

### 3.2 Oxidative Stress and Neuroinflammation Impair Microglial Phagocytic Function

Building on the observation of early microglial activation, we further elucidate how sustained Aβ phagocytosis contributes to an oxidative extracellular environment that induces neurotoxicity. The persistent activation of microglia results in increased intracellular ROS levels and the formation of an oxidative extracellular microenvironment, which collectively impair microglial membrane integrity and function. Additionally, the elevated Ca^2+^ influx activates the NF‐*κ*B pathway (nuclear localization of phosphorylated‐NF‐*κ*B) and promotes actin remodeling,^[^ ^40^ ^]^ exacerbating inflammation and neurotoxicity during the Aβ clearance process (Figure S10, Supporting Information). These molecular events underscore the detrimental effects of chronic microglial activation, where oxidative stress and neuroinflammation synergistically impair the ability of microglia to effectively clear Aβ aggregates, thereby contributing to AD progression.

### 3.3 Resveratrol Mitigates Oxidative Stress and Enhances Microglial Phagocytic Activity

RSV, a potential antioxidant with anti‐inflammatory properties, is identified as a favorable therapeutic agent in our study. RSV effectively mitigates both intracellular and extracellular ROS levels in microglia during the early and middle stages of Aβ clearance, as demonstrated by reduced ROS fluorescence and decreased H_2_O_2_ oxidation currents (Figure 3B,C). Furthermore, RSV treatment prevents neurotoxicity induced by oxidative stress, thereby sustaining the integrity of the microglial cell membrane (Figure 3D). Importantly, RSV sustains the expression of Trem2, a key regulator of microglial phagocytosis, thereby enhancing the phagocytic activity of microglia even at the late stages of Aβ clearance (Figure 3E,F). Our findings suggest that RSV not only reduces the oxidative and inflammatory burden on microglia but also enhances their functional capacity to clear Aβ aggregates (Figure 5B), highlighting its potential as a therapeutic strategy to improve microglial function and slow AD progression.

### 3.4 Innovative Use of SECM and SICM Provides Real‐Time Insights into Microglial Function

Our study introduces the innovative application of SECM and SICM to monitor real‐time changes in ROS levels and phagocytic activity of microglia during Aβ clearance. These techniques enable us to dynamically track extracellular H_2_O_2_ production and the movement of Aβ oligomers on the microglial cell membrane with high spatial and temporal resolution. The integration of SECM and SICM provides a comprehensive understanding of the mechanobiological processes underpinning microglial activation and dysfunction in AD. This methodological advancement not only enhances our ability to study microglial behavior in real‐time but also offers broader implications for investigating cellular processes in other neurodegenerative diseases. Our findings complement in vivo studies of how microglia sense and respond to Aβ stiffness to regulate the inflammatory microenvironment. Additionally, the results provide valuable spatiotemporal insights into the dynamics of oxidative stress and phagocytosis during Aβ clearance.

### 3.5 Implications for Alzheimer's Disease Therapeutics and Future Directions

Our findings underscore the critical role of mechanotransduction in microglial activation, revealing that the mechanical stiffness of Aβ plaques directly induces activation through the PIEZO1 mechanosensing pathway. This mechanistic insight identifies the PIEZO1 signal pathway for modulating microglial activation and neuroinflammation in AD. Additionally, the temporal dynamics of ROS production, where extracellular H_2_O_2_ precedes intracellular ROS and pro‐inflammatory cytokine release, highlight the importance of early intervention strategies targeting extracellular ROS to prevent downstream neuroinflammatory cascades. The effective use of RSV in sustaining microglial phagocytic activity while reducing oxidative stress and inflammation presents a promising therapeutic avenue for enhancing microglial function and slowing AD progression. Future research can be extended to in vivo studies exploring the therapeutic potential of targeting mechanotransduction pathways and examining the role of microglia in AD pathogenesis, which will help deepen our understanding of how microglial responses to Aβ plaques contribute to disease progression. And to validate the efficacy of antioxidants like RSV in clinical settings, we can use localized delivery techniques and nano‐delivery systems to improve brain penetration and bioavailability of RSV in in vivo models, enhancing RSV's therapeutic potential by specifically targeting microglia to activate Aβ clearance for more effective treatment in AD models.^[^ ^41^, ^42^ ^]^ In addition, constructing in vivo models that reflect the neuroinflammatory environment associated with Aβ plaques in AD still faces challenges, such as the lack of coordinated interactions between microglia and other brain cells (e.g., astrocytes and neurons) and interference from abnormally activated microglia.^[^ ^43^, ^44^, ^45^ ^]^ Future studies combining in vitro Aβ plaque‐associated models with co‐culture systems can shed light on how these interactions influence microglial Aβ phagocytosis and neuroinflammation. Finally, the methodological advancements demonstrated in this study pave the way for more detailed investigations into the biophysical and biochemical interactions governing microglial behavior in neurodegenerative diseases.

In conclusion, our study integrates biophysical and biochemical analyses to unravel the complex interplay between Aβ plaque mechanics, microglial activation, and neuroinflammation. By identifying novel therapeutic targets and demonstrating the efficacy of resveratrol, we provide valuable insights that may inform future strategies for the treatment of AD.

## 4 Experimental Section

### Preparation of Polyacrylamide (PA) Gels

The PA gels for BV2 cells were prepared following a previous report.^[^ ^46^ ^]^ Briefly, the PA gels with varying stiffness were prepared by adjusting the ratios of 40% (w/v) acrylamide, 2% (w/v) *N*, *N‘*‐methylene‐bis‐acrylamide, 10% (w/v) ammonium persulfate, and 1% (w/v) *N*, *N*, *N*′, *N*′‐tetramethylethylenediamine. To prepare the PA gels with stiffness of ≈150 and 600 Pa, the ratios of acrylamide (%)/*N*, *N′*‐methylene‐bis‐acrylamide (%) were adjusted to 5/0.045, 6/0.050, and the volumes of ammonium persulfate and *N*, *N*, *N*′ *N*′‐tetramethylethylenediamine were kept at 1/100 and 1/1000 of the total volume, respectively. The premixed solution (45 µL) was then sandwiched between the glass slides (NEST, 18 mm) of the glass bottom dishes (NEST, 35 mm) and the glass coverslips, which were modified with 200 µL 2% 3‐(trimethoxysilyl) propyl methacrylate and dichloromethylsilane to ensure the PA gel attachment to the glass slides and easy detachment of coverslips after polymerization. After polymerization for 15 min under room temperature, the PA gels and coverslips were immersed in PBS for 20 min, and then the coverslips were removed to obtain the PA gels. The exposed PA gels were rinsed in PBS for three times. Each as‐prepared PA gel was treated with 300 µL hydrazine hydrate for functionalization for 12 h. The gels were then treated with 5% acetic acid solution for 1 h, followed by washing with sterile PBS for three times. The as‐prepared PA gels were exposed to UV light at 365 nm for 45 min for sterilization. Finally, poly‐*D*‐lysine solution was added to the PA gel surfaces for 30 min at 37 °C to ensure conjugation with PA gels to promote cell adhesion, followed by washing with sterile PBS for three times. The stiffness of the PA gels was characterized by a Piuma Nanoindenter (Optics 11, Netherlands).

### Preparation of Aβ Plaques

Unlabeled and FAM‐labeled Beta‐Amyloid (1‐42) (Aβ_1‐42_) (Anaspec Inc.) were stored at −80 °C. For Aβ monomers preparation, Aβ powder was dissolved with DMSO at a concentration of 1 mm. Subsequently, Aβ plaques were prepared by diluting the Aβ monomers to 100 µm in DMEM and then incubated at PA gels with 37 °C for 72 h to promote fibrillation.

### Cell Culture and Aβ Incubation

BV2 cells (Cell Culture Center of Institute of Basic Medical Sciences, Chinese Academy of Medical Sciences, China) were cultured with DMEM medium supplemented with 10% fetal bovine serum, 1% HEPES, 1% GlutaMAX‐1 and 1% penicillin/streptomycin in an incubator (5% CO_2_, 37 °C). BV2 cells were cultured on the 156.2 Pa PA gels at a density of 5 × 10^4^ cm^−2^ and incubated in an incubator (5% CO_2_, 37 °C) for 24 h. Subsequently, these BV2 cells were digested with trypsin and seeded on 575.7 Pa PA gels with Aβ plaques at a density of 5 × 10^4^ cm^−2^. Last, the cells were incubated for 2–24 h at 37 °C and 5% CO_2_ before all experiments.

### Theoretical Model of SECM

The membrane permeability of BV2 cells on the PA gels during the Aβ clearance process was analyzed by fitting the approach curves of SECM experiments with the theoretical model. The SECM simulation model was developed in a 2D axisymmetric coordinate system using COMSOL Multiphysics 5.5 (COMSOL Inc., Sweden). As illustrated in Figure S3A (Supporting Information), the *r*‐axis and *z*‐axis are parallel and perpendicular to a Pt‐modified carbon microelectrode surface (0.5 µm in radius, *RG* = 1.4, where *RG* is the ratio of the overall electrode radius and the radius of carbon), respectively. The origin of the coordinate axes was positioned at the center of the Pt‐modified carbon microelectrode.

Based on the previous work,^[^ ^21^ ^]^ the definitions of the domain, diffusion, and model boundary of the redox medium were defined (Table S1, Supporting Information). As depicted in Figure S3B (Supporting Information), the bulk solution and the cell were divided as two separate regions and the diffusion of [Ru(NH_3_)_6_]Cl_3_ in both regions follows Fick's second law of diffusion, as described by Equations (1) and (2).

(1) $$\frac{\partial C_{B}\left(r,z,t\right)}{\partial t}=D\left(\frac{\partial^{2}C_{B}\left(r,z,t\right)}{\partial r^{2}}+\frac{1}{r}\frac{\partial C_{B}\left(r,z,t\right)}{\partial r}+\frac{\partial^{2}C_{B}\left(r,z,t\right)}{\partial z^{2}}\right)$$

(2) $$\frac{\partial C_{C}\left(r,z,t\right)}{\partial t}=D\left(\frac{\partial^{2}C_{C}\left(r,z,t\right)}{\partial r^{2}}+\frac{1}{r}\frac{\partial C_{C}\left(r,z,t\right)}{\partial r}+\frac{\partial^{2}C_{C}\left(r,z,t\right)}{\partial z^{2}}\right)$$

where *C*_B_ and *C*_C_ are the concentrations of [Ru(NH_3_)_6_]Cl_3_ outside and inside of cell, *t* is the time, *r* and *z* are the axisymmetric coordinates, and *D* is the diffusion coefficient of [Ru(NH_3_)_6_]Cl_3_ in advanced Tyrode's solutions at 37 °C (*D* = 1.3 × 10^−5 ^cm^2^ s^−1^ in this case).^[^ ^47^ ^]^

The boundary of the cell membrane could be defined as the flux (Equations (3) and (4)). The coefficient (*P*_m_) was defined as the speed of [Ru(NH_3_)_6_]Cl_3_ across the cell membrane, which was driven by the difference in concentration between the bulk solution and the cell.

(3) $$f_{in}=P_{m}\left(C_{B}-C_{C}\right)$$

(4) $$f_{out}=P_{m}\left(C_{C}-C_{B}\right)$$

The concentration [Ru(NH_3_)_6_]Cl_3_ around the Pt‐modified carbon microelectrode was obtained by integrating the flux on the surface of the Pt‐modified carbon microelectrode at each simulated electrode position. The current on the Pt‐modified carbon microelectrode surface could be calculated by Equation (5).

(5) $$\;i=2\pi \mathrm{nDF}\underset{0}{\int^{a}}r\left[\frac{\partial C_{B}\left(r,z,t\right)}{\partial z}\right]\mathrm{dr}$$

where *n* is the electron transfer number (*n *= 1 in this case), *F* is the Faraday constant (96 485 C mol^−1^), and *a* is the SECM probe radius (*a* = 0.5 µm in this case). The model geometry was finer meshed before computation. The cell geometry in the SECM experiments, the parameterized probe position, and *P*_m_ were used to obtain the theoretical approach curves by the parametric sweep function of COMSOL. By adapting the *P*_m_ value and the distance between the SECM probe and the cell surface, the theoretical approach curves with varied membrane permeability around the cell surface could be obtained.

### Statistical Analysis

Statistical comparisons were performed with GraphPad Prism 9.2.0 and Origin 9.0.

## Conflict of Interest

The authors declare no conflict of interest.

## Author Contributions

F.L., F.X., J.Z., and Y.L.L. designed the study. Y.L.L., J.J.Z., F.X.F., and Q.Q.A. performed the SECM and SICM measurements, collected and analysed the data. Y.L.L., Y.X.Z., and S.Y.Z. conducted the cell experiments and data analysis. F.L., F.X., J.Z., and Y.L.L. prepared the manuscript. All authors discussed the experiments, read and commented on the manuscript.

## Supporting information



Supporting Information

## Data Availability

The data that support the findings of this study are available in the supplementary material of this article.
