# Supplementary material for: Mechanotransduction Activates Microglia and Impairs Phagocytosis in Stiff Amyloid‐β Plaques
Source: Adv Sci (Weinh). 2025 May 23;12(30):e03389. doi: 10.1002/advs.202503389 (PMC12376493; doi:10.1002/advs.202503389)
Supplement: Supplementary file 1 — Supporting Information [file ADVS-12-e03389-s001.docx]

**Supporting Information**

**Mechanotransduction Activates Microglia and Impairs Phagocytosis in Stiff Amyloid-β Plaques**

Yulin Liu,^1,2^ Junjie Zhang,^1,2^ Yuxiang Zhao,^1,2^ Feixiang Fang,^1,2^ Siyu Zhang,^1,2^ Qiqi An,^1,2^ Jian Zhuang,^3*^ Feng Xu,^1,2*^ and Fei Li^1,2*^

*^1^ The Key Laboratory of Biomedical Information Engineering of Ministry of Education, School of Life Science and Technology, Xi’an Jiaotong University, Xi’an 710049, P. R. China*

*^2^ Bioinspired Engineering and Biomechanics Center (BEBC), Xi’an Jiaotong University, Xi’an 710049, P. R. China*

*^3^ Key Laboratory of Education Ministry for Modern Design Rotor-Bearing System, School of Mechanical Engineering, Xi’an Jiaotong University, Xi’an 710049, P.R. China*

*^*^Corresponding authors: zhuangjian@mail.xjtu.edu.cn; fengxu@mail.xjtu.edu.cn; feili@mail.xjtu.edu.cn*

**Table of contents**

1. [**Experimental section** S3](#_Toc133785907)

[1.1 Chemicals and reagents S3](#_Toc133785908)

[1.2 Western blot analysis S3](#_Toc133785908)

[1.3 Characterizations of TNF-α, IL-1β, IL-6, NO and ROS levels of BV2 cells S4](#_Toc133785912)

[1.4 Preparations and characterizations of SECM and SICM probes S4](#_Toc133785913)

[1.5 SECM and SICM experiments S5](#_Toc133785914)

**2. Parameters of SECM theoretical model**  S6

[Table S1 S7](#_Toc133785917)

**3. Supporting figures** [S7](#_Toc133785923)

[Figure S1. S7](#_Toc133785924)

[Figure S2. S7](#_Toc133785925)

[Figure S3. S8](#_Toc133785926)

[Figure S4 S8](#_Toc133785927)

[Figure S5 S9](#_Toc133785928)

[Figure S6 S9](#_Toc133785929)

[Figure S7 S9](#_Toc133785929)

[Figure S8 S10](#_Toc133785929)

[Figure S9 S11](#_Toc133785929)

[Figure S10 S11](#_Toc133785929)

[**References** S11](#_Toc133785930)

1. Experimental section

1.1 Chemicals and reagents

Hexaammineruthenium (III) chloride ([Ru(NH_3_)_6_]Cl_3_), acrylamide (Acr), N, N′ methylene-bis-acrylamide (MBA), ammonium persulfate (APS), N, N, N′, N′-tetramethylethylenediamine (TEMED), chloroplatinic acid, sodium sulphate and poly-D-lysine were all purchased from Sigma-Aldrich Inc. (U.S.A.). Human Aβ_1-42_ peptides (AS-20276) and FAM-Aβ_1-42_ (AS-23526-01) were purchased from Anaspec Inc. (U.S.A.). Resveratrol and GsMTx4 were obtained from MedChemExpress LLC (U.S.A.). High-glucose Dulbecco’s modified Eagle’s medium (HG-DMEM) was obtained from Gibco Life Technologies Inc. (U.S.A.). Penicillin/streptomycin, trypsin and advanced Tyrode's solution were all obtained from Procell Co., Ltd. (China). Fetal bovine serum (FBS) and phosphate buffered saline (PBS) were purchased from InCellGene LLC (U.S.A.). Mouse TNF-α, IL-1β, IL-6 ELISA kits, ATP assay kit, BCA protein assay kit, Fluo-4 Ca^2+^ kit and Griess reagent kit were obtained from Beyotime Biotechnology Co., Ltd. (China). Live/dead kits, Alexa Fluor™ 555 Phalloidin and pHrodo™ Red *E. coli* BioParticles were purchased from Thermo Fisher Scientific Inc. (U.S.A.). ROS/superoxide detection assay kit, anti-PIEZO1 antibody (ab259949), anti-Trem2 (ab305103), anti-NOX2/gp91phox antibody (ab129068), anti-SYK (ab40781), and goat anti-rabbit/anti-mouse IgG H&L (Alexa Fluor® 488/594) were all obtained from Abcam PLC (U.K.). DAPI and RIPA lysis buffer were purchased from Solarbio Science and Technology Co., Ltd. (China). Anti-Phospho-Zap-70 (Tyr319)/Syk (Tyr352), Anti-Akt (pan) (C67E7) and Phospho-Akt (Ser473) were all obtained from Cell Signaling Technology, Inc. (U.S.A.). All the solutions used in cell experiments were filtered with 0.22-μm membranes (Millex-GP, Germany) before use. The aqueous solutions used in this work were all made from a Milli-Q water purification system (Millipore, resistivity >18.2 MΩ, U.S.A.).

1.2 Western blot analysis

BV2 cells were firstly cultured on the PA gels deposited with or without Aβ plaques at a density of 5×10^4^ cells cm^−2^, incubated in an incubator (5% CO_2_, 37 °C) for 12 h, and then lysed by RIPA lysis buffer. The total protein concentrations of BV2 cells were determined using a BCA protein assay kit. The protein samples of BV2 cells were subjected to 15% sodium dodecyl sulfate-polyacrylamide gel electrophoresis and transferred to PVDF membranes. Thereafter, the PVDF membranes were blocked with 1.5% BSA for 2 h and incubated with anti-Trem2, anti-SYK, anti-pSYK, anti-AKT and anti-pAKT primary antibodies, respectively. After 12 h, the membranes were stained with HRP-conjugated secondary antibody for 2 h under room temperature. Finally, the membranes were visualized and measured using a chemiluminescence imaging system (Clinx, 3300 mini, China).

1.3 Characterizations of TNF-α, IL-1β, IL-6, NO and ROS levels of BV2 cells

The BV2 cells were inoculated on the PA gels with stiffness about 156.2 Pa at a density of 5×10^4^ cells/well. After culturing for 24 h, the BV2 cells were digested and reseeded on 575.7 Pa PA gels deposited with Aβ plaques at a density of 5×10^4^ cells/well, and then incubated for 2, 4, 6, 8, and 12 h, respectively. The expressions of TNF-α, IL-1β, IL-6, the concentration of NO and the intracellular ROS levels of BV2 cells were measured with TNF-α, IL-1β, IL-6, NO and ROS/superoxide detection assay kits, respectively, following the manufacturer's instructions. A multifunctional microplate reader (SPARK 10 M, TECAN, Switzerland) was used to record the absorbance of these inflammatory factors at 450 nm. The LIVE/DEAD staining and the intracellular ROS levels of BV2 cells were characterized with a confocal microscope (Olympus FV3000, Nikon, Japan).

1.4 Preparations and characterizations of SECM and SICM probes

The Pt-modified carbon microelectrodes used as the SECM probes were prepared following the previous report.^[1]^ In brief, a quartz glass capillary (Q100-70-10, Sutter Instruments, USA) was pulled using a laser puller (Model P-2000, Sutter Instruments, USA) using the following parameters: Heat, 565; Fil, 4; Vel, 60; Del, 145; Pull, 175. The carbon microelectrode was prepared after heating the pulled glass pipettes filled with methylbenzene with a Bunsen burner for 5 s with milling for controlling the disk size and the geometry of carbon. Then the Pt-modified carbon microelectrodes were fabricated by electrochemical deposition of Pt on the exposed carbon microdisk surface with applying a potential range of 0.1 V to −0.6 V (vs. Ag/AgCl RE) in an aqueous solution containing 1 mM H_2_PtCl_6_ and 0.2 mol L^-1^ sodium sulfate.^[2]^ The electrochemical performance of the prepared carbon microelectrodes and the Pt-modified carbon microelectrodes were tested by cyclic voltammetry (CV) in 0.1 M KCl solution containing 1 mM [Ru(NH_3_)_6_]Cl_3_.

For preparation of the SICM probes, the nanopipettes with a typical radius of 15-20 nm were drawn from quartz glass capillaries (Q100-70-10, ID; Sutter Instruments, USA) using a Laser Puller P-2000 (Sutter Instruments, USA) using the following parameters: Step I: Heat, 750; Fil, 4; Vel, 30; Del, 150; Pull, 80, Step II: Heat, 650; Fil, 3; Vel, 40; Del, 135; Pull, 180. Then the fabricated nanopipettes were filled with an advanced Tyrode’s solution as used in the bath for the following SICM experiments.

1.5 SECM and SICM experiments

***SECM experiments***

The SECM instrument (ElProScan PG618, HEKA Elektronik GmbH, Harvard Bioscience Inc.) integrated with an inverted fluorescence microscope (Olympus-IX53, Olympus Co., Ltd., Japan) was used for all the SECM experiments. SECM measurements were performed using a three-electrode system consisting of 1-μm diameter Pt-modified carbon microelectrode (*RG* = 1.4, where *RG* is the ratio of the overall electrode radius and the radius of carbon) as the working electrode, and a 0.6-mm-in-diameter Ag/AgCl wire and a 0.5-mm-in-diameter platinum wire as the reference electrode (RE) and counter electrode (CE), respectively. To ensure the precise control of the temperatures during SECM experiments, a heated incubator (TC-344C, Warner Instruments) was combined with the SECM system to sustain the cell culture at a constant temperature around 37.0±0.1°C. The whole temperature-controlled SECM platform was placed in a Faraday cage and well grounded.

Before SECM experiments, the BV2 cells were seeded on the PA gels at a density of 5×10^4^ cells/well, and placed in an incubator (5% CO_2_, 37 °C) for 2, 4, 8 and 12 h, respectively. Then the culture medium of BV2 cells was replenished with an advanced Tyrode’s solution containing 1 mM [Ru(NH_3_)_6_]Cl_3_ and equilibrated for 15 min. Subsequently, the SECM probe with an applied potential of −0.35 V (vs. Ag/AgCl RE) was approached to the cell surface, raised by another 1 μm and moved to the cell edge. The probe currents across the BV2 cell surface along the *x-*axis and *y-*axis directions with the fixed *z*-direction were recorded to acquire the highest position of the cell.

For monitoring of the H_2_O_2_ generated from the BV2 cells on the PA gels after incubation of Aβ plaques for 2, 4, 8 and 12 h, the SECM probe was first approached to the BV2 cell surface from about 20 μm and then positioned 1 μm above the highest point of the cell. Subsequently, a potential of 0.65 V (vs. Ag/AgCl RE) was applied to the SECM probe and the currents of H_2_O_2_ oxidation were recorded.

For characterization of the membrane permeability of BV2 cells on the PA gels, the SECM probe applied with a potential of −0.35 V (vs. Ag/AgCl RE) was firstly placed from about 20 μm above the BV2 cell surface, and then approached to the highest point of the cell with an approach velocity of 0.2 μm s^−1^. Subsequently, the SECM approach curves were recorded and the membrane permeability values of BV2 cells on the PA gels with Aβ plaques for 4, 8 and 12 h were obtained by simulating the experimental SECM approach curves with the theoretical model.

***SICM experiments***

The SICM instrument (custom-built) integrated with an optical microscope (Ti2, Nikon Co., Ltd., Japan) was used for all our SICM experiments. The SICM instrument included a nanopipette, a current amplifier (SR570, Stanford Research), an XY piezo positioner (P621.2CL, Physik Instrumente), and a Z piezo positioner (P621.ZCL, Physik Instrumente) for precisely XYZ positioning, which were all controlled by a controller (E-509, Physik Instrumente) and mounted on an anti-vibration table. The SICM measurements were performed using a 30-40 nm nanopipette as the probe, and two 0.3-mm diameter Ag/AgCl electrodes were inserted into the nanopipette and the bath as the reference electrodes, respectively. The SICM system was located with a Faraday cage for minimizing mechanical vibration and electrical noise.

Before SICM experiments, the BV2 cells were seeded on the PA gels deposited with Aβ plaques at a density of 5×10^4^ cells/well, and placed in an incubator (5% CO_2_, 37°C) for 12 h. Then the culture medium of BV2 cells was replenished with an advanced Tyrode’s solution and equilibrated for 15 min. For characterization of the Aβ oligomers on the microglia membrane surface, the 30-40 nm nanopipette was filled with an advanced Tyrode’s solution, two Ag/AgCl electrodes were inserted into the nanopipette and the bath, respectively. Subsequently, the SICM probe applied with a potential of 0.1 V (vs. Ag/AgCl RE) was approached to the cell surface at a rate of 50 μm s^−1^ and a 2% decrease in the ionic current. Lastly, the hopping mode of SICM was used to scan across BV2 cells with scan areas of 2 μm×2 μm.

2. Parameters of SECM theoretical model

**Table S1**. Main experimental parameters used in the SECM 2D simulation model

| **Parameter** | **Unit** | **Value** |
| --- | --- | --- |
| *a* | μm | 0.4-0.75 |
| *RG* | - | 1.2-1.44 |
| *C*_R1_ | mmol | 1 |
| *T* | K | 310.0 |
| *D*_Ru(NH3)6Cl3_ | cm^2^ s^−1^ | 1.30×10^−5^ |
| *h*_cell_ | μm | 7.5-13.0 |
| *r*_cell_ | μm | 7.5-13.0 |

3. Supporting figures


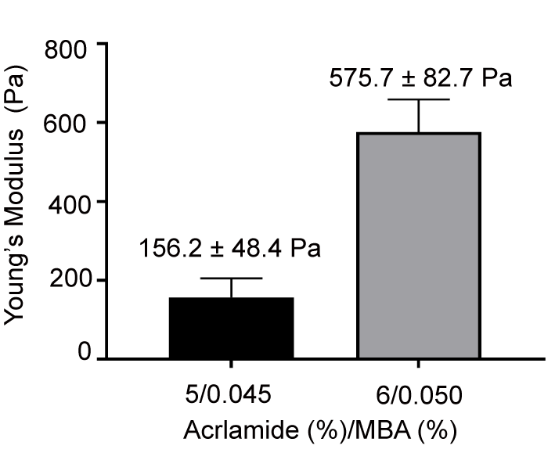


Figure S1. Young’s modulus of the as-prepared PA gels (*n* = 28).


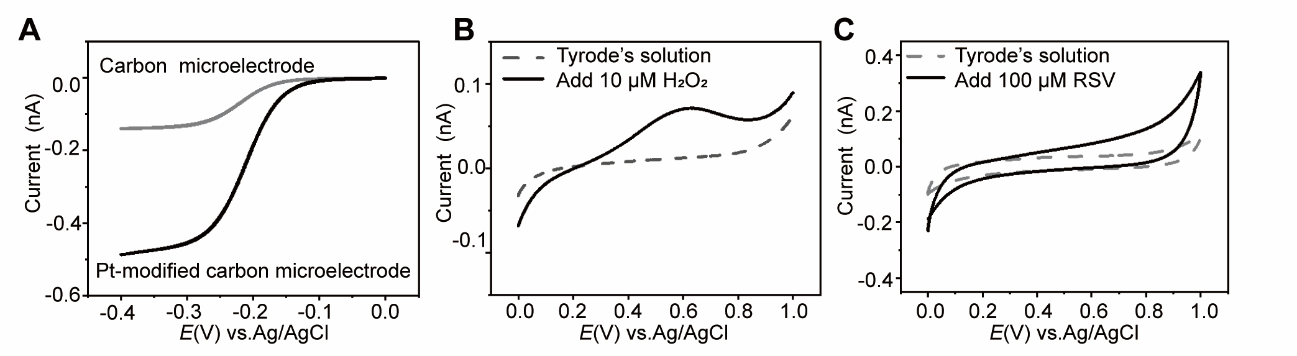


Figure S2. (A) Cyclic voltammograms in 0.1 M KCl solution containing 1 mM [Ru(NH_3_)_6_]Cl_3_ using a carbon microelectrode or a Pt-modified carbon microelectrode as the working electrode (scan rates: 10 mV s^-1^); (B) Linear sweep voltammograms in the advanced Tyrode’s solution without (dotted curve) and with adding 10 μM H_2_O_2_ (black curve) using a Pt-modified carbon microelectrode as the working electrode; (C) Cyclic voltammograms recorded at a Pt-modified carbon microelectrode in the advanced Tyrode’s solution with and without adding 100 μM RSV. A three-electrode system with a 1 μm-in-diameter carbon microelectrode or a Pt-modified carbon microelectrode as the working electrode, a 0.6 mm-in-diameter Ag/AgCl wire as the reference electrode and a 0.5 mm-in-diameter platinum wire as the counter electrode was used (scan rates: 10 mV/s).


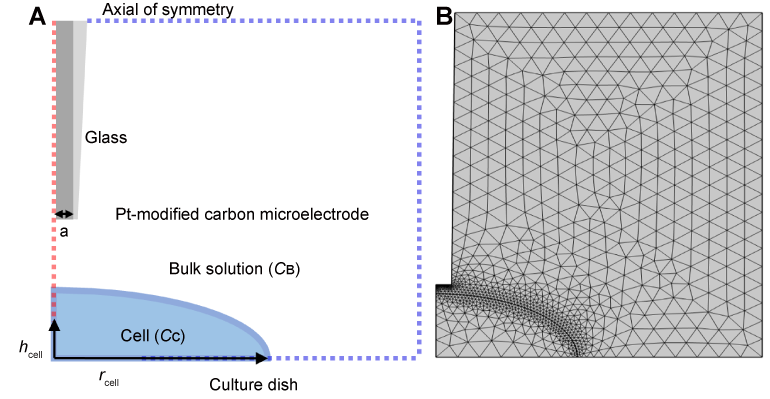


Figure S3. (A) Schematic diagram of SECM theoretical model in the 2D axial symmetry and (B) diagram of mesh refinement in the Pt-modified carbon microelectrode and cell membrane.

**
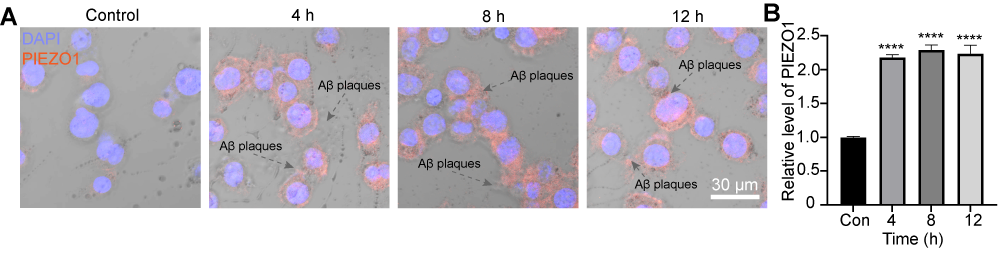
**

Figure S4. Characterization results of PIEZO1 expressions in BV2 cells on 575.7 Pa PA gels during the Aβ clearance process. (A) Immunofluorescence images of PIEZO1 expressions in BV2 cells at 24 h and after incubation of Aβ plaques for 4, 8 and 12 h, respectively; (B) Analysis of PIEZO1 expression levels in (A). Statistical significance: *****p*<0.0001 (one-way ANOVA).

**
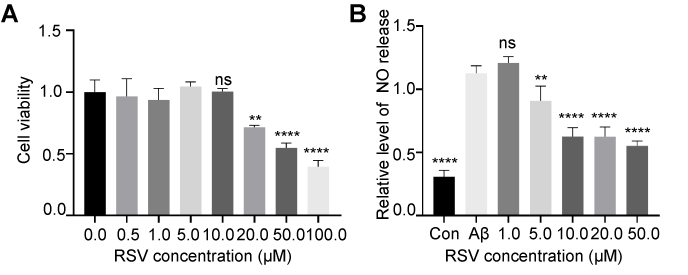
**

Figure S5. (A) Cell viability of BV2 cells on 575.7 Pa PA gels after adding 0.0, 0.5, 1.0, 5.0, 10.0, 20.0, 50.0 and 100.0 μM RSV, respectively; (B) Relative levels of NO release of BV2 cells after incubation of Aβ plaques with adding 1.0, 5.0, 10.0, 20.0 and 50.0 μM RSV, respectively, for 12 h (*n* = 3). Statistical significance: *ns*, no signiﬁcant diﬀerence, ***p*<0.001, *****p*<0.0001 (one-way ANOVA).


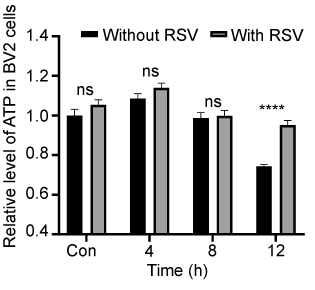


Figure S6. Characterization results of relative levels of ATP in BV2 cells on 575.7 Pa PA gels after incubation with Aβ plaques for 4, 8 and 12 h, respectively, with or without RSV treatment (*n* = 3). Statistical significance: *ns*, no signiﬁcant diﬀerence, *****p*<0.0001 (one-way ANOVA).

**
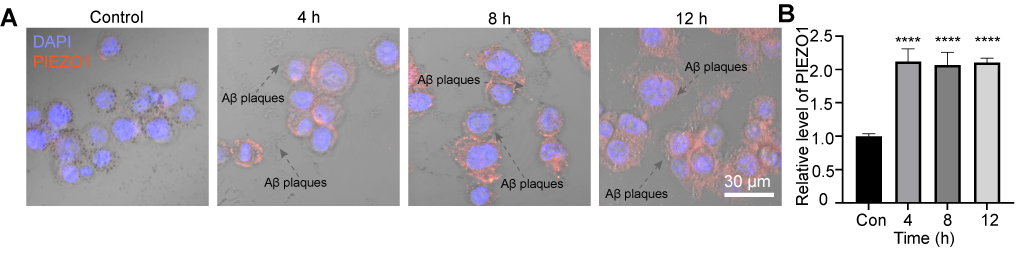
**

**Figure S7. Characterization results of** **PIEZO1 expressions in BV2 cells on 575.7 Pa PA gels during the Aβ clearance process with RSV treatment.** (A) Immunofluorescence images of PIEZO1 expressions in BV2 after incubation of Aβ plaques for 4, 8 and 12 h, respectively, with RSV treatment; (B) Analysis of PIEZO1 expression levels in BV2 cells in (A). Statistical significance: *****p*<0.0001 (one-way ANOVA).


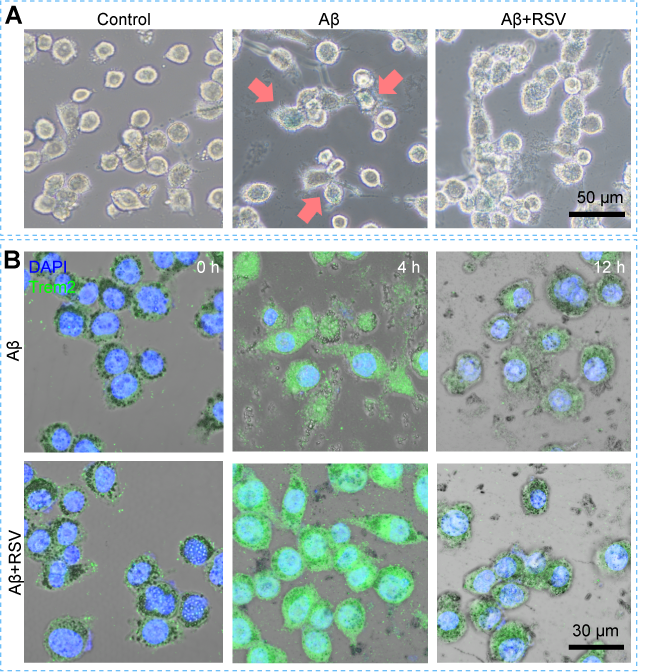


Figure S8. Characterization results of aging-associated SA-β-Gal activity and Trem2 expression in BV2 cells on 575.7 Pa PA gels during the Aβ clearance process. (A) Optical microscope images of aging-associated SA-β-Gal activity of BV2 cells on the PA gels after incubation of Aβ plaques with and without RSV treatment for 12 h, respectively (red arrowheads depict SA-*β*-Gal activity); (B) Immunofluorescence images of Trem2 expressions in BV2 cells on the PA gels after incubation of Aβ plaques with and without RSV treatment for 0, 4 and 12 h, respectively.


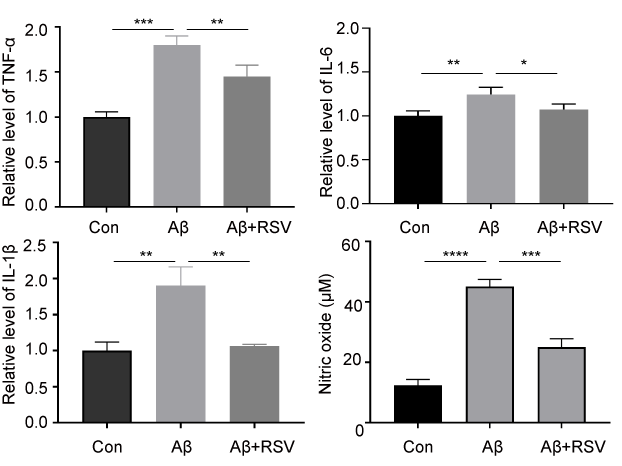


Figure S9. Characterization results of relative TNF-α, IL-6, IL-1β expressions and NO concentrations of BV2 cells on 575.7 Pa PA gels deposited with Aβ plaques with and without RSV treatment for 12 h (*n* = 3). Statistical significance: **p*<0.05, ***p*<0.001, ****p*<0.0005 and *****p*<0.0001 (one-way ANOVA).


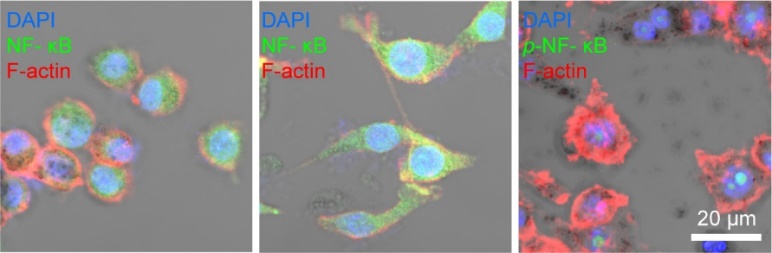


Figure S10. Immunofluorescence images of NF-*κ*B and phosphorylated-NF-*κ*B (*p*-NF-*κ*B) in BV2 cells on 575.7 Pa PA gels after incubation with Aβ plaques for 12 h.

References

1. Y. Liu, J. Zhang, Y. Li, Y. Zhao, S. Kuermanbayi, J. Zhuang, H. Zhang, F. Xu, F. Li. Matrix stiffness-dependent microglia activation in response to inflammatory cues: in situ investigation by scanning electrochemical microscopy. *Chem. Sci.* **2024**, *15*, 171-184.
2. F. Zhang, Q. Qiu, X. Yang, W. Huang. Real-time electrochemical detection of retrograde messengers. *Chinese J. Anal. Chem.* **2019**, *47*, 6.
